# Knowledge, attitude, and practices of veterinarians towards canine vector-borne pathogens in Sri Lanka

**DOI:** 10.1371/journal.pntd.0012365

**Published:** 2024-07-29

**Authors:** Ushani Atapattu, Vito Colella, Rebecca J. Traub, Anke Wiethoelter

**Affiliations:** 1 Melbourne Veterinary School, Faculty of Science, University of Melbourne, Victoria, Australia; 2 Department of Infectious Diseases and Public Health, City University of Hong Kong, Hong Kong SAR, China; UDLA: Universidad de Las Americas, ECUADOR

## Abstract

Canine vector-borne pathogens (CVBP) have a worldwide distribution and show a high prevalence in tropical countries such as Sri Lanka. Some CVBP are zoonotic, with dogs identified as reservoir hosts for human subcutaneous dirofilariasis and potentially for spotted fever rickettsioses and re-emergent brugian filariasis in Sri Lanka, making these pathogens emerging public health issues in the country. Veterinarians are crucial in monitoring, preventing, and controlling these pathogens in dogs. Therefore, it is imperative to understand veterinarians’ knowledge, attitude, and practices (KAP) regarding CVBP to mitigate their impact. A survey was designed and administered electronically to veterinarians residing and practising in Sri Lanka. Responses were evaluated using descriptive, univariable, and multivariable analyses to investigate associations between demographic factors, knowledge, attitude, and practices related to CVBP. Out of the 170 participating veterinarians, nearly 70% had moderate or high knowledge. However, the awareness of zoonotic pathogens, *Brugia* spp. (16%) and *Rickettsia conorii* (18%), was low, and a considerable number of veterinarians were unaware of the zoonotic nature of *Dirofilaria repens*. Based on multivariable analysis adjusting for experience and self-rated knowledge, new graduates had higher odds of knowledge compared to experienced veterinarians (OR 5.7, 95% CI 1.7–23, p = 0.028). Questions assessing the attitude towards CVBP indicated that most participating veterinarians comprehend and agree with their importance. Nearly all participants agreed that ectoparasite control is the best option to prevent CVBP infections (91%, 153/167) and that for effective treatment of CVBP, a definitive diagnosis is required (81%, 135/167). However, veterinarians recommended suboptimal treatments for some CVBP, like *Babesia gibsoni*. Better practices were associated with being a companion animal practitioner (OR 2.4, 95% CI 1.1–5.7, p = 0.032) and having a low to moderate canine caseload (OR 3.6, 95% CI 1.3–10.4, p = 0.038). Limited knowledge of zoonotic CVBP among veterinarians, along with suboptimal treatment, might contribute to dogs acting as reservoirs and high prevalence of these pathogens in Sri Lanka. Therefore, continued veterinary education is recommended to improve knowledge and practices, which in turn will help to improve the diagnosis, treatment, and control of these infections in Sri Lanka to ensure the well-being of dogs and humans.

## Author summary

Pathogens spread by ticks, fleas, and mosquitos, so called vector-borne pathogens, are common in tropical countries like Sri Lanka. Some vector-borne pathogens affect dogs as well as humans, potentially causing severe illness in both. As dogs can act as reservoir hosts, preventing these infections is vital for animal and human health. Veterinarians are the experts when it comes to diagnosing, treating, and controlling vector-borne pathogens in dogs. Thus, their understanding and approach to these diseases are key in reducing their impact. This study highlights both the strengths and gaps in veterinarians’ knowledge, attitude and practices concerning vector-borne pathogens and identifies areas for improvement. This will assist relevant stakeholders and policymakers to develop targeted continuing veterinary education and best practice guidelines for diagnosis, treatment, and prevention of diseases caused by vector-borne pathogens in dogs. This will not only lead to better care for dogs affected by these diseases, but ultimately aid in reducing the risk to human health.

## Introduction

Vector-borne pathogens (VBP) infecting domestic dogs (*Canis lupus familiaris*) are widespread throughout tropical Asia [1]. These pathogens include bacterial species such as *Ehrlichia canis* and *Anaplasma platys*, as well as protozoan parasites such as *Babesia* spp. and *Hepatozoon canis* [1–4], with ticks being primarily responsible for their transmission. Dogs may have a role as reservoir for rickettsioses caused by several species of the genus *Rickettsia* [5], such as *Rickettsia conorii* [6] and *Rickettsia felis* [7]. Mosquitoes can also act as vectors of pathogens, such as filarial nematodes in the genera *Brugia* and *Dirofilaria*, which infect dogs and can pose a risk to humans [6,8–10].

In Sri Lanka, canine vector-borne pathogens (CVBP) are ubiquitous, with a high prevalence of *Babesia gibsoni* and *H*. *canis* [11] and a lesser but considerable prevalence of *A*. *platys*, *E*. *canis*, *Babesia vogeli*, and haemotropic mycoplasma species [11]. Furthermore, the high prevalence of zoonotic filariae in pet dogs such as *Dirofilaria* sp. ‘hongkongensis’ and *Brugia* sp. Sri Lanka genotype is contributing to an emerging public health issue [12,13], with Sri Lanka recently reporting the second-highest number of human subcutaneous dirofilariasis cases in the world [14]. Dogs in Sri Lanka have also been found to be seropositive for typhus and spotted fever rickettsioses (SFR) [15], which currently cause severe diseases in humans in Sri Lanka [16–19].

Sound knowledge and practices around CVBP are essential for veterinarians as they are involved in the diagnosis, treatment, and prevention of infections caused by these pathogens [20–23]. Currently, no information is available on veterinarians’ knowledge, attitude and practices (KAP) around CVBP in Sri Lanka. This is a shortcoming in understanding what knowledge level is present, what recommendations are made, what practices are used, and whether those practices effectively mitigate the impact of these pathogens. A convenient and efficient method to assess this is KAP surveys [24], which have been used in health and health-related sectors. For example, several studies explored veterinarians’ KAP related to zoonoses, canine vector-borne diseases, and One Health [22,25–30]. This study aimed to obtain an overview of KAP of veterinarians to provide a better understanding of potential enablers and barriers to the implementation of effective and efficient diagnosis, treatment, and control of CVBP in Sri Lanka.

## Materials and methods

### Ethics statement

This study was approved by the Office of Research Ethics and Integrity at the University of Melbourne (Reference Number 2021-20764-15614-3).

### Target population

The majority of veterinarians in Sri Lanka graduate from the Faculty of Veterinary Medicine and Animal Sciences at the University of Peradeniya (https://vet.pdn.ac.lk/), which is the only veterinary school in the country. To be able to work in Sri Lanka, veterinarians must meet the required qualifications according to the Veterinary Surgeons and Practitioners Act [31] and register with the Veterinary Council of Sri Lanka (VCSL). As of August 2020, 2041 veterinarians have been registered under the VCSL since the early 1950s, indicating the maximum possible size of the veterinary workforce in Sri Lanka [32].

### Survey design and data collection

Since veterinarians in Sri Lanka receive their education in English and are competent in the language, a KAP survey was designed and conducted in English using the Research Electronic Data Capture (REDCap) platform [33,34]. The survey was open to all veterinarians residing and registered to practice in veterinary or public health-related fields in Sri Lanka and is provided in **S1 File**. It comprised two main sections: the first section included core questions around demographic characteristics, knowledge, and attitude towards CVBP for all veterinarians. Demographic information collected included age, gender, graduation year, veterinary college, and primary discipline of practice. Data on attitude were collected on a 5-point Likert scale using statements around veterinary and public health implications and the importance of CVBP. The second part of the survey targeted canine practitioners, defined as veterinarians who either engaged exclusively in companion animal practice and treated dogs or any veterinarian who indicated that at least a quarter of their routine caseload consisted of dogs. Practices around diagnosis, treatment, and control of CVBP by these veterinarians were collected.

Seven volunteers tested the survey to ensure all questions were clear and easily understood prior to distributing the survey through social media (e.g., Facebook) and instant messaging platforms (e.g., WhatsApp and Viber). Informed written consent was obtained from each participant. Survey responses were collected from April to November 2021.

### Data analysis

Data were downloaded and cleaned using Microsoft Excel for Microsoft 365 MSO (Version 2212) and R version 4.2.0 in R studio Desktop (version 2023.3.1.446) [35] with ‘dplyr’ [36] and ‘janitor’ [37] packages. Responses less than 75% of the core questions were deemed incomplete and excluded from subsequent analysis. Explanatory variables were visualised and assessed using frequencies, bar charts and contingency tables utilising the packages ‘janitor’ [37], ‘sjPlot’ [38] and ‘DescTools’ [39]. Categorical variables were created for CVBP information sources (≤3, 4, ≥5) and experience level based on graduation year (≥ 2019 –new graduate, 2014–2018 –moderately experienced, ≤ 2013 –experienced). Responses were collapsed into three categories for the variables, canine caseload (≤ 50% –no-moderate, 51–75% –high, >75%–very high), confidence of Sri Lankan veterinarians handling CVBP (low, moderate, high) and self-rated knowledge on CVBP (low, moderate, high).

Using answers to 11 knowledge questions (**S1 File,** Questions 13–24), a knowledge score was calculated by allocating two points for each correct answer, one point for "don’t know" using it as a proxy for self-awareness of a knowledge gap, and zero points for incorrect answers. Knowledge of endemic VBP in Sri Lanka was assessed using a checklist (**S1 File,** Question 24), with one point allocated for each pathogen correctly identified as present or absent as of December 2021. The knowledge score was then categorised into "low" (≤ 25 points), "medium" (26–29), and "high" (≥ 30) and used as a response variable in subsequent uni- and multivariable analyses.

For canine practitioners, a practice score was calculated using seven questions around CVBP diagnosis (**S1 File,** Question 39), treatment (**S1 File,** Questions 40, 43–46), and prevention (**S1 File,** Question 42). Five-point Likert scale responses were collapsed with zero points allocated to "never" or "rarely", one point to "sometimes", and two points to "very often" or "always". Using the most effective drug for treatment based on peer-reviewed literature [40–42] was allocated two points, while other treatments were allocated zero points. The resulting CVBP practice score ranged from 0–14 points. It was categorised into "low-moderate" (≤ 9) and "high" (> 9) and used as a response variable for subsequent uni- and multivariable analyses.

Associations between demographic factors, attitude, and knowledge were investigated with ordinal regression using the ‘MASS’ package [43]. Logistic regression using the ‘stats’ package was applied to investigate associations between CVBP practices and attitude, knowledge and demographics. Likelihood P-values were obtained through the ‘emmeans’ package [44]. The variables, experience level, age, primary discipline, confidence in the profession, and knowledge scores were further collapsed as "new graduates or moderately experienced" and "experienced", "25–34 years" and "≥ 35 years", "companion animal practice" and "other disciplines", and "low-moderate" and "high", respectively to accommodate the limited number of participating canine practitioners.

A concept map, including ecological, socioeconomic and professional factors [45,46], was created to identify potential influences on the practices of Sri Lankan veterinarians around CVBP. To identify potential associations between explanatory variables such as demographics of the veterinarians and knowledge as well as practices around CVBP, directed acyclic graphs (DAGs) were constructed using DAGitty version 3.0 [47]. Those informed multivariable analyses.

Variables for multivariable models were selected based on univariable associations with at least marginal significance (p ≤ 0.25) and were fitted using backwards-stepwise elimination. The independence of model terms was evaluated by calculating pairwise correlation coefficients with Cramer’s V test using ‘creditmodel’ package [48]. Coefficients ≥ 0.6 indicated high correlation, and only one of the variables was then included in the analysis. Confounding was investigated by adding variables individually back into the final model and assessing whether the model coefficients changed by ≥ 20%. This would suggest that the added variable substantially influences the relationship between the independent and response variables and thus acts as a confounder. For the adjusted ordinal regression model, adherence to the assumption of proportional odds was checked using the ‘brant’ package [49] and the model fit was evaluated using the Lipsitz goodness of fit test for ordinal responses [50] with the ‘generalhoslem’ package [51]. For the adjusted logistic regression model, the fitness of the model was validated using diagnostic plots (residuals versus fitted values, normal quantile-quantile, and residuals versus leverage). All illustrations used in this publication were constructed using Adobe Illustrator version 27.5 (Adobe Inc. USA).

## Results

Overall, 206 veterinarians responded to the survey invite, and 170 completed surveys were included in the analysis. Using data of veterinarians registered to practice in Sri Lanka [32], this relates to a response proportion of 8.3% (170/2041).

### Demographics of participants

Demographic details of the responding veterinarians (n = 170) are summarised in **Table 1.** Most veterinarians were females (58%, 99/170), 25–34 years old (75%, 128/170) and moderately experienced (59%, 101/170). All participants, except one, graduated from the University of Peradeniya. More than 70% (124/170) of the respondents were either involved in companion animal practice or were government veterinarians. While 84% (143/170) stated that they encounter dogs as patients, only 75% (128/170) either specifically focused on small animals or had a canine caseload of at least 25% and thus met our definition of canine practitioner.

**Table 1:** Demographic and knowledge details of Sri Lankan veterinarians participating in a survey on knowledge, attitudes, and practices around canine vector-borne pathogens (CVBP) (n = 170).

Variable	Category	n	%
Gender	Female	99	58.2
	Male	67	39.4
	Unknown	4	2.4
Age group (years)	25–34	128	75.3
	35–44	25	14.7
	45–54	12	7.1
	≥ 55	5	2.9
Veterinary college	University of Peradeniya	169	99.4
	Other†	1	0.6
Experience	New graduates (≥ 2019)	16	9.4
	Moderately experienced (2014–2018)	101	59.4
	Experienced (≤ 2013)	53	31.2
Primary discipline of practice	Companion animal practice	66	38.8
	Government	58	34.1
	Academia	32	18.8
	Other^‡^	14	8.2
Species encountered	Dogs	143	84.1
	Cats	131	77.1
	Avian species (including poultry)	87	51.2
	Ruminants	77	45.3
	Wildlife species	37	21.8
	Swine	35	20.6
	Equine	10	5.9
	Rodents & rabbits	4	2.4
	None	4	2.4
	Fish	2	1.2
Canine caseload	No or low (0–24%)	44	25.9
	Moderate (25–50%)	36	21.2
	High (51–75%)	49	28.8
	Very High (>75%)	41	24.1
Canine practitioner	Yes	128	75.3
	No	42	24.7
Information sources used to update knowledge on CVBP	Textbooks	133	78.2
	Colleagues	125	73.5
	Other sources on the internet	107	62.9
	Publications	103	60.6
	Teachers/ academics	80	47.1
	Webinar	50	29.4
	Social media	35	20.6
	Workshops	34	20
	No sources	3	1.8
No. of information sources used	≤ 3	72	42.4
	4	38	22.3
	≥ 5	60	35.3
VBP knowledge score	Low	56	32.9
	Moderate	76	44.7
	High	38	22.4
Self-rated CVBP knowledge	Low	6	3.5
	Moderate	73	42.9
	High	91	53.5
Agreement between self-rated vs. actual knowledge	Underestimation	29	17.1
	Accurate estimation	91	53.5
	Overestimation	50	29.4

^†^Madras Veterinary College

^‡^Includes farm animal practice, poultry, wildlife, and exotics

### Knowledge on CVBP

The preferred method of Sri Lankan veterinarians to obtain information on CVBP was through textbooks (78%, 133/170) and colleagues (74%, 125/170), with 42% (72/170) using a maximum of three different types of information sources. Most veterinarians (>85%) were aware of the definition and concepts of CVBP (**S1 Table**). Nevertheless, only 52% responded correctly stating the vector of *Trypanosoma evansi* as flies, and 57% indicated correctly that *D*. *repens* infects humans (**S1 Table**). Over 70% of the veterinarians could correctly identify 10 of the 15 VBP listed as either present or absent in Sri Lanka (**S2 Table**). However, only 12%, 16%, 18%, and 32% were aware of the presence of haemotropic mycoplasmas, spotted fever *Rickettsia* (*R*. *conorii*), filariae of the genus *Brugia*, and *A*. *platys*, respectively. Overall, 33% (56/170) of Sri Lankan veterinarians had low CVBP knowledge scores, while 45% (76/170) had medium, and 22% (38/170) had high knowledge scores (**Table 1**). When asked to self-rate their knowledge on CVBP, 46% assessed their knowledge as low or moderate, while the remaining 54% regarded their knowledge as high. Comparing self-assessed and actual knowledge, 53% accurately gauged their knowledge level, while 29% overestimated and 17% underestimated their understanding of CVBP (**Table 1**).

### Attitude related to CVBP

**Fig 1:**
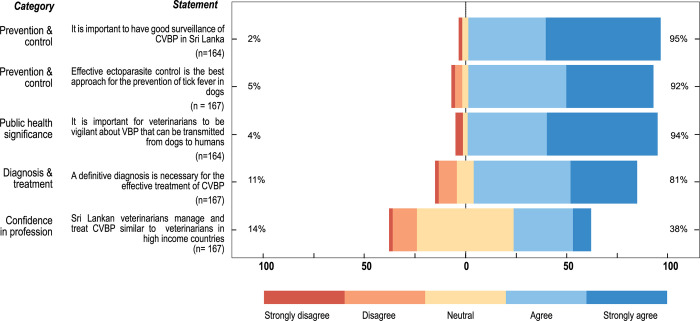
Responses to statements evaluating attitude towards diagnosis, treatment, prevention, control, public health implications, and confidence in profession, regarding canine vector-borne pathogens (CVBP) of veterinarians in Sri Lanka.

**Fig 1** shows the responses of veterinarians to attitude-related statements. Most statements were met with high levels of agreement. Over 50% of veterinarians strongly agreed that good surveillance for CVBP (56%, 92/164) and vigilance regarding zoonotic CVBP (54%, 89/164) were important. Nearly all veterinarians (153/167) agreed that ectoparasite control is the best option to prevent CVBP infections, while over 80% (135/167) agreed that for effective treatment of CVBP, a definitive diagnosis is required. However, a greater proportion remained neutral (48%, 79/164) regarding their confidence in the profession to treat and manage CVBP infections in a similar fashion to high-income economies.

### Practices around CVBP infections by canine practitioners

The practices related to diagnosis, treatment, and prevention of CVBP by canine practitioners are described in **S3 Table**. Tick fever (babesiosis and ehrlichiosis) was the most frequently encountered CVBP infection, with 64% (81/126) of canine practitioners encountering daily cases, followed by filariasis (33%, 41/126). For trypanosomiasis, more than half of the respondents (52%, 65/126) reported that they had never diagnosed a case. Furthermore, the majority of veterinarians stated to experience good to excellent prognosis for tick fever (95%, 117/123) and filariasis (81%, 99/122). However, nearly half of the veterinarians were unaware of the prognosis of canine trypanosomiasis (49%, 60/123). While 66% (78/118) of canine practitioners utilised laboratories or other diagnostic facilities very often or always to diagnose CVBP, 43% (51/118) based their diagnosis on clinical signs. Monetary constraints when diagnosing CVBP were encountered very often or always by 32% (38/118) of the participating canine practitioners. When treating canine babesiosis, most canine practitioners preferred imidocarb dipropionate (42% for *B*. *gibsoni*, 48% for *B*. *vogeli)*, while the preferred choice for canine ehrlichiosis was doxycycline (66%). For dirofilariasis, macrocyclic lactones (50%) and levamisole (41%) were indicated as the preferred choices.

Most canine practitioners (85%, 100/118) stated that they always inform dog owners about ectoparasite control, with topical fipronil (76%), systemic isoxazolines (63%), and propoxur powders (51%) as the products of choice to control tick, flea, and louse infestations. Overall, 54% (64/118) of canine practitioners had low to moderate CVBP practice scores, and 46% (54/118) had high CVBP practice scores (**S3 Table)**.

### Factors associated with knowledge and practices around CVBP

**Fig 2:**
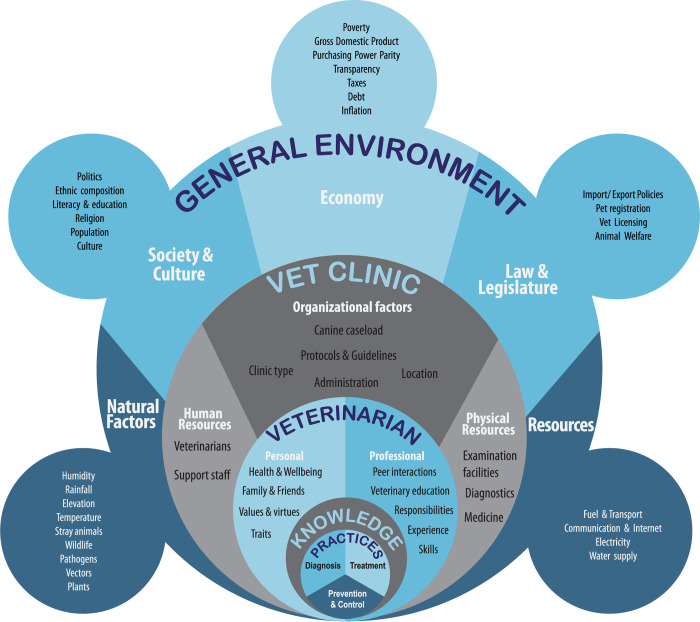
Concept map identifying the interplay of ecological, socioeconomic and professional factors potentially influencing the knowledge, attitude, and practices of Sri Lankan veterinarians around canine vector-borne pathogens.

**Fig 3:**
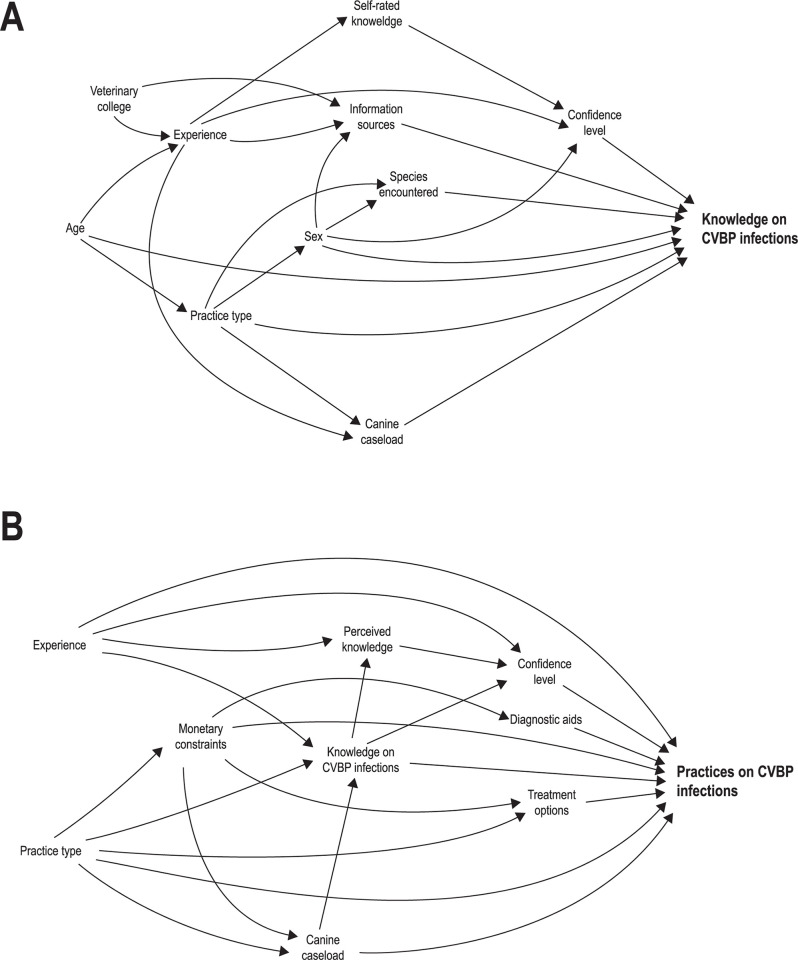
Simplified directed acyclic graphs (DAG) depicting potential associations with A) knowledge of Sri Lankan veterinarians on canine vector-borne pathogens (CVBP) and B) practices of veterinarians in Sri Lanka related to CVBP infections.

A concept map identifying the complex interplay of macroenvironmental factors and those of the immediate environment that can directly or indirectly influence the veterinarians’ knowledge, attitude, and practices related to CVBP is illustrated in **Fig 2**. Factors pertaining to nature, society and culture, economy, law and legislature, and resources were identified and reflect the general environment. Nested within the general environment are factors of the immediate environment, such as those associated with the clinic/hospital and the veterinarian themselves (**Fig 2**). While all these factors influence the diagnosis, treatment, and control of CVBP, not all of them can be changed in order to achieve improvements. Furthermore, not all influencers can be measured and quantified. Consequently, two DAGs depicted in **Fig 3A** and **Fig 3B** were created, simplifying and delineating the associations between veterinarians’ knowledge and the practices of canine practitioners related to CVBP, respectively.

In univariable analysis, gender, age, experience, primary discipline of practice, number of information sources used to update knowledge and self-rated knowledge were associated (p ≤ 0.25) with the knowledge of CVBP (**S4 Table**). These variables were included in the initial multivariable ordinal regression model to determine factors associated with CVBP knowledge. As age and experience level of veterinarians were highly correlated, only experience level was included in the multivariable analysis. **Table 2** shows factors associated with CVBP knowledge of veterinarians in Sri Lanka. Females had three times higher odds of knowledge compared to their male colleagues (OR 3, 95% CI 1.6–5.7, p < 0.001). Odds of knowledge in new graduates were six times higher (OR 5.7, 95% CI 1.7–23, p = 0.028) compared to experienced veterinarians. Those who rated their knowledge as high had higher odds of CVBP knowledge (OR 2, 95% CI 1.1–3.7, p = 0.028) (**Table 2**). When testing the final model, no confounders were identified as the coefficients of the final model from backward step-wise elimination did not change by ≤ 20% when variables were reintroduced. The assumption of proportional odds was fulfilled (p > 0.05), and the Lipsitz goodness of fit test indicated a sufficient model fit (p = 0.79).

**Table 2:** Final multivariable ordinal regression model for factors associated with knowledge on canine vector-borne pathogens of veterinarians in Sri Lanka (n = 166).

Variables	Estimate	SE	Odds Ratios (95% CI)	P-value
Intercept Low	-0.09	0.37		
Intercept High	1.73	0.39		
**Gender**				<0.001
Female	1.11	0.32	3 (1.6–5.7)	
Male			Reference	
**Experience**				0.028
New graduate	1.74	0.65	5.7 (1.7–23)	
Moderately experienced	0.29	0.33	1.3 (0.7–2.5)	
Experienced			Reference	
**Self-rated knowledge**				0.028
Low—moderate			Reference	
High	0.68	0.31	2 (1.1–3.7)	

CI = confidence interval; SE = standard error

Univariable associations showed that practices around diagnosing, treating, and preventing CVBP were associated (p ≤ 0.25) with the primary discipline of practice, the number of information sources used to update knowledge, and the canine caseload (**S5 Table**) and were included in the initial multivariable logistic regression model. The final multivariable logistic regression model evaluating practices of canine practitioners around CVBP in Sri Lanka indicated that veterinarians engaged exclusively in companion animal practice had higher odds of following best practices (OR 2.4, 95% CI 1.1–5.7, p = 0.032) than those involved in other practice types such as academia and government sector (**Table 3**). In addition, veterinarians presented with a low-moderate canine caseload had higher odds of having higher practice scores (OR 3.6, 95% CI 1.3–10.4, p = 0.038) compared to those who encountered very high canine caseloads (**Table 3**). No confounders were identified as the coefficients of the final model from backward stepwise elimination did not change by ≤ 20% when variables were reintroduced. In addition, diagnostic plots indicated a good model fit.

**Table 3:** Final multivariable logistic regression model for factors associated with the treatment, diagnosis, and prevention practices around canine vector-borne pathogen infections of canine practitioners in Sri Lanka (n = 118).

Variables	Estimate	SE	Odds Ratios (95% CI)	P-value
Intercept	-1.11	0.44		
**Primary discipline of practice**				0.032
Companion animal practice	0.89	0.41	2.4 (1.1–5.7)	
Other^†^			Reference	
**Canine caseload**				0.038
Low—moderate (≤ 50%)	1.28	0.52	3.6 (1.3–10.4)	
High (>50%—≤ 75%)	0.26	0.46	1.3 (0.5–3.3)	
Very high (>75%)			Reference	

^†^includes academia, government, wildlife, and exotics; CI = confidence interval; SE = standard error

## Discussion

This is the first comprehensive analysis of KAP related to CVBP of veterinarians in Sri Lanka. It provides valuable insights into how CVBP are diagnosed, treated, and prevented under the local context and highlights several avenues for continued veterinary education (CVE) to improve knowledge and practices. Such focused improvement can aid in uplifting veterinary services and formulating control protocols for CVBP, which are crucial to mitigate the veterinary and zoonotic impact of these pathogens.

In the present study, 67% of participating veterinarians had moderate or high knowledge scores. Compared to similar studies conducted with veterinary professionals in the states of Illinois and Ohio in the USA [22,25] and Mongolia [26], Sri Lankan veterinarians seem to be knowledgeable on CVBP. For instance, 75% of veterinarians in Illinois were only able to answer 10% of the knowledge questions on CVBP correctly [25], whereas, in this survey, 75% of the veterinarians in Sri Lanka answered 70% of the knowledge questions correctly. Only 43% of veterinarians in Ohio were able to correctly identify that *Ehrlichia* is present in their state [22], while the presence of *Ehrlichia* was correctly identified by 97% of Sri Lankan veterinarians. Furthermore, over half of the respondents in Mongolia had not heard of certain CVBP [26]. However, it is important to note that with their continental climate and cold winters [52], Illinois, Ohio and Mongolia have a lesser risk of CVBP burden compared to the tropical climate in Sri Lanka, making CVBP knowledge a vital component in the routine practice for Sri Lankan veterinarians.

Although over 75% of Sri Lankan veterinarians gave correct responses to nearly 70% of the knowledge questions in the survey, less than half responded correctly to questions on zoonotic VBP, demonstrating low awareness around this group of pathogens. Despite the notable incidence of human subcutaneous dirofilariasis caused by *D*. *repens* (recently genetically characterised [12] as *Dirofilaria* sp. ‘hongkongensis’) [53–59] and its high prevalence in dogs in Sri Lanka [12,60], only 57% of veterinarians identified this pathogen as a risk to humans. However, this proportion is higher compared to Baltic and Nordic countries, where only 34% of veterinarians considered this pathogen to be zoonotic [61]. Nevertheless, low awareness of *Brugia* species among Sri Lankan veterinarians is concerning as *Brugia* [62] in humans is re-emerging, and domestic dogs seem to act as reservoirs [12,60,63]. Similarly, dogs are known reservoirs of *R*. *conorii* in Sri Lanka [15]. Nevertheless, hardly 20% of Sri Lankan veterinarians were aware that *R*. *conorii* is endemic to Sri Lanka, which is considerably lower compared to veterinary students in Brazil (84%) [64]. Awareness of zoonotic VBP is not only crucial for veterinary professionals to minimise occupational health risks but also to mitigate their impact on human and animal health [64]. Thus, a lack of knowledge among veterinarians could directly threaten the effective control of these pathogens [13,65,66].

The multivariable analysis identified females, new graduates, and those who self-rated their CVBP knowledge as high to have higher odds of knowledge. A comparable trend was evident from similar studies evaluating the knowledge, attitude and practices of VBP among veterinarians in Mongolia and the USA [22,26]. New graduates are likely to possess fresh and most up-to-date knowledge compared to older, more experienced veterinarians. Furthermore, Sri Lankan veterinarians appear to be accurate in their self-evaluation of knowledge. Similarly, veterinarians from Ohio, USA, also correctly rated their knowledge [28], possibly signifying that veterinarians in general have a good conscience about their level of knowledge.

The attitude-evaluating statements demonstrated that Sri Lankan veterinarians had a high agreement on the importance of preventing and controlling VBP through surveillance and ectoparasite control. A similar high agreement on the importance of zoonotic tick-borne diseases was also observed with veterinarians in Ohio, USA [28]. Such a positive attitude potentially supports veterinarians’ willingness to mitigate the impact of these pathogens [22].

In addition to knowledge and attitude, we were able to provide an overview of practices related to CVBP diagnosis, treatment, and control in Sri Lanka. In concordance with the results of previous studies indicating a high prevalence of CVBP in Sri Lanka [11,12,60,67], veterinarians in this study reported frequently encountering CVBP cases such as tick fever and filariasis. Nearly half of the participating veterinarians stated that they would diagnose CVBP using only clinical signs without the aid of other diagnostics. A study of field veterinarians in Sri Lanka also indicated the infrequent use of diagnostic laboratories [68]. One possible explanation for the use of inadequate diagnostics could be monetary constraints encountered, as indicated by a considerable proportion of participants. In contrast, in Ohio, USA, over 70% of veterinarians employed external laboratory facilities to identify tick species discovered in their patients [22]. Not using adequate diagnostics can lead to incorrect diagnosis, as clinical signs of most CVBP are non-specific [69]. For example, inadequate diagnostic practices most likely caused less frequent diagnoses of *H*. *canis* despite its high prevalence [11]. Consequently, this could prevent the administration of appropriate treatment and the total clearance of the pathogen, thereby facilitating the emergence of reservoirs for CVBP. This is concerning as some of these pathogens are zoonotic, or potentially could infect animals of economic importance and vulnerable wildlife.

Evaluating treatment practices holistically, some of the CVBP were treated using substandard treatment protocols. For instance, most canine practitioners preferred imidocarb dipropionate to treat *B*. *vogeli* and *B*. *gibsoni*, even though its efficacy towards *B*. *gibsoni* is low to none [70]. While these substandard protocols may improve the patient clinically, their reduced efficacy may only lead to partial elimination of the pathogen, potentially creating reservoirs of infection and leading to relapses [71]. In addition, some treatments can cause adverse reactions [70]. For example, diminazene aceturate is effective against *B*. *gibsoni*, but its variable therapeutic index in dogs can lead to severe toxic manifestations [72], sometimes outweighing its therapeutic benefits. Most veterinarians recommended effective ectoparasiticides, with fipronil-based products being the preferred choice. However, when comparing the veterinarians’ recommendations to the products administered by dog owners [11], they do not seem to comply with the recommendations made. A probable explanation might be the higher cost of the recommended products, as most dog owners mentioned cheaper but less effective options as their preferred choices [11].

Companion animal practitioners and those who reported to encounter a low to moderate proportion of canine cases had higher odds of better practices. They might be driven to portray better practices around CVBP compared to other veterinarians (e.g., academia and the government sector) due to the changing role of dogs in Sri Lankan households over the past few decades [73]. Over 40% of dog owners now consider dogs as companions for themselves and their children [74], making them an integral part of their families. An increase in dog ownership and status invariably increases the demand for specialised veterinary services [73]. While one might expect increasing canine caseloads to lead to better practices, in this study, the best practices were observed by those who encountered low to moderate canine caseloads. One possible explanation for this might be that specialised companion animal veterinarians encounter not only dogs but also cats, pet birds, and pocket pets, thereby reducing their proportion of canine cases seen. On the other hand, government veterinarians performing anti-rabies vaccination and dog neutering [75] might have a very high proportion of canine cases.

Several limitations apply to this study. The total number of registered veterinarians [32] includes those who are out of practice, overseas, or deceased, likely decreasing our target population size and increasing the actual proportion responded. In addition, COVID-19 pandemic related events that took place during the surveying period potentially influenced the number of responses received. Nevertheless, the resulting response rate is low and subject to selection bias. In addition, having the survey exclusively online might have led to lower participation of less technology-savvy veterinarians. The high proportion of female participants could be explained by the feminisation of the veterinary workforce as demonstrated in several other studies [76–79]. Veterinarians having an interest in the topic might have been more likely to complete the questionnaire. Hence, participants likely represent an engaged, best-informed cohort regarding CVBP knowledge and practices in Sri Lanka. However, knowledge gaps and suboptimal practices were present among this cohort which highlights the necessity of CVE to develop good standards of knowledge and practices around CVBP in Sri Lanka. Another limitation of this study is that it is largely based on self-reported behaviour and therefore prone to measurement bias. Knowledge and practices around CVBP are subject to an intricate interplay of macroenvironmental influences, encompassing economic, ecological, legislative, and societal factors [80,81]. These factors are not only hard to measure but also challenging to alter. This study focused foremost on modifiable factors, even though they are not the sole contributors.

## Conclusions

Conclusively, veterinarians in Sri Lanka responded correctly to most knowledge questions on CVBP and have a positive attitude towards controlling and preventing these infections. However, their awareness of the zoonotic CVBP, drawbacks in diagnosis and treatment needs improvement. Therefore, we recommend raising awareness and providing opportunities for continuing education for veterinarians towards improving the veterinary diagnostic and treatment facilities, particularly in the government sector, to increase the quality and accessibility of veterinary services offered in relation to CVBP in Sri Lanka. Implementing cost-effective diagnostic protocols for effective CVBP diagnosis and drugs for treating and preventing these pathogens should be re-evaluated to apprehend deficiencies. Formulation of national standards for best practices on CVBP under varying local contexts would foster improved diagnosis, treatment, and control of these infections due to increased compliance by both the veterinarian and the dog owner. Strengthening cross-disciplinary communication and involvement, especially between medical and veterinary professionals, would strengthen the control of zoonotic CVBP. Holistically, these practices would contribute towards the ensuring good health and well-being of both animals and humans on the island.

## Supporting information

S1 File Questionnaire administered to veterinarians to obtain data on knowledge, attitude, and practices on CVBP. (PDF)

S2 File Data obtained from the knowledge, attitudes, and practices questionnaire on CVBP for Sri Lankan veterinarians. (XLSX)

S1 Table Responses provided by veterinarians in Sri Lanka (n = 170) to knowledge evaluation statements regarding canine vector-borne pathogens. (PDF)

S2 Table Vector-borne pathogens endemic to Sri Lanka according to the scientific literature up until December 2021, and a summary of responses obtained from Sri Lankan veterinarians through a knowledge, attitude, and practices survey around canine vector-borne pathogens. (PDF)

S3 Table Responses of canine practitioners in Sri Lanka on diagnosis, treatment, control, and the prognosis of CVBP infections in dogs. (PDF)

S4 Table Demographic factors, confidence in profession and self-rated knowledge associated with knowledge around canine vector-borne pathogen infections determined through univariable ordinal regression based on responses of veterinarians in Sri Lanka for a knowledge, attitude, and practices survey. (PDF)

S5 Table Associations of knowledge, practice type, confidence in profession, experience, canine caseload, and monetary constraints with practices on diagnosis, treatment, and control of canine vector-borne pathogen infections determined through univariable logistic regression based on responses of canine practitioners in Sri Lanka for a knowledge, attitude, and practices survey. (PDF)

## References

[1] Colella V, Nguyen VL, Tan DY, Lu N, Fang F, Zhijuan Y, et al. Zoonotic vector-borne pathogens and ectoparasites of dogs and cats in Eastern and Southeast Asia. Emerg Infect Dis. 2020;26(6):1221−33. doi: 10.3201/eid2606.191832 32441628 PMC7258489

[2] Huggins LG, Colella V, Koehler AV, Schunack B, Traub RJ. A multipronged next-generation sequencing metabarcoding approach unearths hyperdiverse and abundant dog pathogen communities in Cambodia. Transboundary Emer Dis. 2021. doi: 10.1111/tbed.14180 34096687

[3] Manoj RRS, Iatta R, Latrofa MS, Capozzi L, Raman M, Colella V, Otranto D. Canine vector-borne pathogens from dogs and ticks from Tamil Nadu, India. Acta Trop. 2020;203:105308. 31862465 10.1016/j.actatropica.2019.105308

[4] Irwin PJ, Jefferies R. Arthropod-transmitted diseases of companion animals in Southeast Asia. Trends Parasitol. 2004;20(1):27−34. doi: 10.1016/j.pt.2003.11.004 14700587

[5] Abdel-Shafy S, Abdullah HHAM, El-Molla A, Salib FA, Ghazy AA. Epidemiology and diagnosis of rickettsioses in animal hosts and tick vectors. Bulgarian Journal of Veterinary Medicine. 2019;22(4):371−98.

[6] Levin ML, Killmaster LF, Zemtsova GE. Domestic dogs (*Canis familiaris*) as reservoir hosts for *Rickettsia conorii*. Vector-Borne and Zoonotic Diseases. 2012;12(1):28−33. 21923270 10.1089/vbz.2011.0684

[7] Ng-Nguyen D, Hii SF, Hoang MT, Nguyen VT, Rees R, Stenos J, Traub RJ. Domestic dogs are mammalian reservoirs for the emerging zoonosis flea-borne spotted fever, caused by *Rickettsia felis*. Sci Rep. 2020;10(1):4151. 32139802 10.1038/s41598-020-61122-y PMC7058065

[8] Simón F, Siles-Lucas M, Morchón R, González-Miguel J, Mellado I, Carretón E, Montoya-Alonso JA. Human and animal dirofilariasis: The emergence of a zoonotic mosaic. Clinical Microbiology Reviews. 2012;25(3):507−44. doi: 10.1128/CMR.00012-12 22763636 PMC3416488

[9] World Health Organization. Lymphatic filariasis 2022 [Available from: https://www.who.int/news-room/fact-sheets/detail/lymphatic-filariasis.

[10] Simon F, Siles-Lucas M, Morchon R, Gonzalez-Miguel J, Mellado I, Carreton E, Montoya-Alonso JA. Human and animal dirofilariasis: the emergence of a zoonotic mosaic. Clin Microbiol Rev. 2012;25(3):507−44. doi: 10.1128/CMR.00012-12 22763636 PMC3416488

[11] Atapattu U, Colella V, Worsley A, Huggins LG, Wiethoelter A, Traub RJ. Prevalence, distribution, and factors associated with vector-borne pathogen infections in pet dogs from different geo-climatic zones in Sri Lanka. Transbound Emerg Dis. 2023;2023:1−21. 10.1155/2023/9467314 PMC12016779 40303684

[12] Atapattu U, Koehler AV, Huggins LG, Wiethoelter A, Traub RJ, Colella V. Dogs are reservoir hosts of the zoonotic *Dirofilaria* sp. ’hongkongensis’ and potentially of *Brugia* sp. Sri Lanka genotype in Sri Lanka. One Health. 2023;17:100625. 38024272 10.1016/j.onehlt.2023.100625 PMC10665175

[13] Mallawarachchi CH, Chandrasena NTGA, Premaratna R, Mallawarachchi SMNSM, de Silva NR. Human infection with sub-periodic *Brugia* spp. in Gampaha District, Sri Lanka: a threat to filariasis elimination status? Parasit Vectors. 2018;11(1):68. 29378620 10.1186/s13071-018-2649-3 PMC5789669

[14] Kini RG, Leena JB, Shetty P, Lyngdoh RH, Sumanth D, George L. Human dirofilariasis: an emerging zoonosis in India. J Parasit Dis 2015;39(2):349−54. doi: 10.1007/s12639-013-0348-8 26064035 PMC4456557

[15] Nanayakkara DM, Rajapakse RP, Wickramasinghe S, Kularatne SA. Serological evidence for exposure of dogs to *Rickettsia conorii*, *Rickettsia typhi*, and *Orientia tsutsugamushi* in Sri Lanka. Vector Borne Zoonotic Dis. 2013;13(8):545−9. 23930973 10.1089/vbz.2012.1049 PMC3741424

[16] Ehelepola NDB, Kumara GDNR, Sapurugala SACS, Buddhadasa WMNP, Dissanayake WP. An atypical case of rickettsial spotted fever myocarditis mimicking Weil’s Disease. Case Reports in Infectious Diseases. 2019;2019:1−6. doi: 10.1155/2019/9620245 31360559 PMC6644265

[17] Herath HMLY, Jayasundara JMHD, Senadhira SDN, Kularatne SAM, Kularatne WKS. Spotted fever rickettsioses causing myocarditis and ARDS: a case from Sri Lanka. BMC Infect Dis. 2018;18(1):705. doi: 10.1186/s12879-018-3631-6 30594148 PMC6311067

[18] Luke N, Munasinghe H, Balasooriya L, Premaratna R. Widespread subcutaneous necrosis in spotted fever group Rickettsioses from the coastal belt of Sri Lanka- a case report. BMC Infect Dis. 2017;17(1). doi: 10.1186/s12879-017-2375-z 28412927 PMC5392909

[19] Weerakoon K, Kularatne SAM, Rajapakse J, Adikari S, Waduge R. Cutaneous manifestations of spotted fever rickettsial infections in the Central Province of Sri Lanka: A descriptive study. PLoS Neglected Tropical Diseases. 2014;8(9):e3179. doi: 10.1371/journal.pntd.0003179 25232837 PMC4169373

[20] Baneth G, Bourdeau P, Bourdoiseau G, Bowman D, Breitschwerdt E, Capelli G, et al. Vector-Borne Diseases—constant challenge for practicing veterinarians: recommendations from the CVBD World Forum. Parasites & Vectors. 2012;5(1):55. doi: 10.1186/1756-3305-5-55 22433172 PMC3359183

[21] Dantas-Torres F, Ketzis J, Mihalca AD, Baneth G, Otranto D, Tort GP, et al. TroCCAP recommendations for the diagnosis, prevention and treatment of parasitic infections in dogs and cats in the tropics. Veterinary parasitology. 2020;283:109167. doi: 10.1016/j.vetpar.2020.109167 32580071

[22] Eleftheriou A, Swisher S, Arruda A, Berrian A, Pesapane R. A survey of knowledge, attitudes, and practices of veterinary professionals regarding ticks and tick-borne diseases: Insights from Ohio, USA. One Health. 2023;17:100592. doi: 10.1016/j.onehlt.2023.100592 37404947 PMC10316080

[23] Wohl JS, Nusbaum KE. Public health roles for small animal practitioners. Journal of the American Veterinary Medical Association. 2007;230(4):494−500. doi: 10.2460/javma.230.4.494 17302544

[24] Siri W, Vivien T, Thaddeus P, Nicole S, D’Arcy R, Amie B. Advocacy, communication and social mobilisation for TB control: a guide to developing knowledge, attitude and practice surveys. Switzerland: WHO Press, World Health Organization; 2008.

[25] Crist SD, Kopsco H, Miller A, Gronemeyer P, Mateus-Pinilla N, Smith RL. Knowledge, attitudes, and practices of veterinary professionals towards ticks and tick-borne diseases in Illinois. One Health. 2022;14:100391. doi: 10.1016/j.onehlt.2022.100391 35686148 PMC9171534

[26] Davitt C, Traub R, Batsukh B, Battur B, Pfeffer M, Wiethoelter AK. Knowledge of Mongolian veterinarians towards canine vector-borne diseases. One Health. 2022;15:100458. doi: 10.1016/j.onehlt.2022.100458 36532679 PMC9754967

[27] James AE, McCall JR, Petersen KR, Wohrle RD, Oltean HN. A survey of veterinarians’ knowledge, attitudes and practices regarding an emerging disease: Coccidioidomycosis in Washington State. Zoonoses and public health. 2020;67(1):25−34. doi: 10.1111/zph.12651 31541564

[28] Jenni ML, Woodward P, Yaglom H, Levy C, Iverson SA, Kretschmer M, et al. Knowledge, attitudes, and practices among veterinarians during an outbreak of canine leptospirosis—Maricopa County, Arizona, 2017. Preventive veterinary medicine. 2019;172:104779. doi: 10.1016/j.prevetmed.2019.104779 31557686

[29] Massetti L, Traub RJ, Rae L, Colella V, Marwedel L, McDonagh P, Wiethoelter A. Canine gastrointestinal parasites perceptions, practices, and behaviours: A survey of dog owners in Australia. One Health. 2023;17:100587. doi: 10.1016/j.onehlt.2023.100587 37415719 PMC10320617

[30] Steele SG, Booy R, Mor SM. Establishing research priorities to improve the One Health efficacy of Australian general practitioners and veterinarians with regard to zoonoses: A modified Delphi survey. One Health. 2018;6:7−15. doi: 10.1016/j.onehlt.2018.08.001 30197925 PMC6127845

[31] Veterinary Surgeons and Practitioners Act No. 46 of 1956 of Sri Lanka.

[32] Veterinary Council of Sri Lanka. The gazette of the Democratic Socialist Republic of Sri Lanka. Colombo2020. p. 1−145.

[33] Harris PA, Taylor R, Thielke R, Payne J, Gonzalez N, Conde JG. Research electronic data capture (REDCap)—A metadata-driven methodology and workflow process for providing translational research informatics support. Journal of Biomedical Informatics. 2009;42(2):377−81. doi: 10.1016/j.jbi.2008.08.010 18929686 PMC2700030

[34] Harris PA, Taylor R, Minor BL, Elliott V, Fernandez M, O’Neal L, et al. The REDCap consortium: Building an international community of software platform partners. Journal of Biomedical Informatics. 2019;95:103208. doi: 10.1016/j.jbi.2019.103208 31078660 PMC7254481

[35] R. Core Team. R: A Language and Environment for Statistical Computing. Vienna, Austria2022.

[36] Wickham H, François R, Henry L, Müller K. dplyr: A Grammar of Data Manipulation. 2022.

[37] Firke S. janitor: Simple Tools for Examining and Cleaning Dirty Data. 2021.

[38] Lüdecke D. sjPlot: Data Visualisation for Statistics in Social Science. 2022.

[39] Signorell A. DescTools: Tools for Descriptive Statistics. R package version 0.99.47 ed2022.

[40] Baneth G. Antiprotozoal treatment of canine babesiosis. Veterinary parasitology. 2018;254:58−63. doi: 10.1016/j.vetpar.2018.03.001 29657012

[41] Bartsch RC, Greene RT. Post-therapy antibody titres in dogs with ehrlichiosis: follow-up study on 68 patients treated primarily with tetracycline and/or doxycycline. Journal of Veterinary Internal Medicine. 1996;10(4):271−4. 8819054 10.1111/j.1939-1676.1996.tb02061.x

[42] Ketzis J, Epe C. Antifilarial chemotherapy: current options in veterinary medicine. Human and Animal Filariases 2022. p. 191−214.

[43] Venables WN, Ripley BD. Modern Applied Statistics with S. 2002.

[44] Lenth RV. emmeans: Estimated Marginal Means, aka Least-Squares Means. 2023.

[45] Shahmanesh M, Harling G, Coltart CEM, Bailey H, King C, Gibbs J, et al. From the micro to the macro to improve health: microorganism ecology and society in teaching infectious disease epidemiology. Lancet Infect Dis. 2020;20(6):e142−e7. doi: 10.1016/S1473-3099(20)30136-5 32386611 PMC7252039

[46] Sokolow SH, Nova N, Jones IJ, Wood CL, Lafferty KD, Garchitorena A, et al. Ecological and socioeconomic factors associated with the human burden of environmentally mediated pathogens: a global analysis. The Lancet Planetary Health. 2022;6(11):e870−e9. doi: 10.1016/S2542-5196(22)00248-0 36370725 PMC9669458

[47] Textor J, van der Zander B, Gilthorpe MS, Liśkiewicz M, Ellison GT. Robust causal inference using directed acyclic graphs: the R package ’dagitty’. Int J Epidemiol. 2017;45(6):1887−94. 10.1093/ije/dyw341 28089956

[48] Fan D. creditmodel: Toolkit for Credit Modeling, Analysis and Visualization. 2022.

[49] Schlegel B, Steenbergen M. brant: Test for Parallel Regression Assumption. 2020.

[50] Lipsitz SR, Buoncristiani JF. A robust goodness-of-fit test statistic with application to ordinal regression models. Statistics in medicine. 1994;13(2):143−52. doi: 10.1002/sim.4780130205 8122050

[51] Jay M. generalhoslem: Goodness of Fit Tests for Logistic Regression Models. R package version 1.3.4 ed2019.

[52] Kottek M, Grieser J, Beck C, Rudolf B, Rubel F. World map of the Köppen-Geiger climate classification updated. Meteorologische Zeitschrift. 2006;15(3):259−63.

[53] Abeysinghe A, Jiffry R, Perera W. Human subcutaneous dirofilariasis: an increasing phenomenon in Sri Lanka. Sri Lanka Journal of Surgery. 2018;36(1).

[54] De Silva GPUP, Udugampola DS, Ekanayake EMIB, Fonseka KVS, Karunadasa MSE. An underrated differential diagnosis for subcutaneous lumps: 26 cases of subcutaneous dirofilariasis. Sri Lanka Journal of Surgery. 2021;39(2):31.

[55] Fernando S, Ihalamulla R, Silva WD. Male and female filarial worms *Dirofilaria (Nochtiella) repens* recovered from the scrotum. Ceylon Medical Journal. 2015;45(3):131. 10.4038/cmj.v45i3.8114 11192993

[56] Iddawela D, Ehambaram K, Wickramasinghe S. Human ocular dirofilariasis due to *Dirofilaria repens* in Sri Lanka. Asian Pac J Trop Med. 2015;8(12):1022−6. 26706673 10.1016/j.apjtm.2015.11.010

[57] Jayasinghe RD, Gunawardane SR, Sitheeque MA, Wickramasinghe S. A Case Report on Oral Subcutaneous Dirofilariasis. Case Rep Infect Dis. 2015;2015:648278. doi: 10.1155/2015/648278 26858848 PMC4697078

[58] Senanayake MP, Infaq MLM, Adikaram SGS, Udagama PV. Ocular and subcutaneous dirofilariasis in a Sri Lankan infant: an environmental hazard caused by dogs and mosquitoes. Paediatrics and International Child Health. 2013;33(2):111−2. doi: 10.1179/2046905512Y.0000000024 23925286

[59] Tilakaratne WM, Pitakotuwage TN. Intra-oral *Dirofilaria repens* infection: report of seven cases. J Oral Pathol Med. 2003;32(8):502−5. 12901735 10.1034/j.1600-0714.2003.00183.x

[60] Mallawarachchi CH, Chandrasena NTGA, Wickramasinghe S, Premaratna R, Gunawardane NYIS, Mallawarachchi NSMSM, et al. A preliminary survey of filarial parasites in dogs and cats in Sri Lanka. PLoS One. 2018;13(11):e0206633. doi: 10.1371/journal.pone.0206633 30388188 PMC6214534

[61] Tiškina V, Jokelainen P. Vector-borne parasitic infections in dogs in the Baltic and Nordic countries: A questionnaire study to veterinarians on canine babesiosis and infections with *Dirofilaria immitis* and *Dirofilaria repens*. Veterinary parasitology. 2017;244:7−11. 28917320 10.1016/j.vetpar.2017.07.012

[62] WHO South-East Asia Regional Office. Recommendations of the Regional Programme Review Group. 2022.

[63] Mallawarachchi CH, Chandrasena TGAN, Withanage GP, Premarathna R, Mallawarachchi SMNSM, Gunawardane NY, et al. Molecular characterisation of a re-emergent *Brugia malayi* parasite in Sri Lanka, suggestive of a novel Strain. Biomed Res Int. 2021;2021:9926101. 34414239 10.1155/2021/9926101 PMC8370822

[64] Barros-Silva PMR, Fonseca LX, Carneiro ME, Vilges KMA, Oliveira SV, Gurgel-Gonçalves R. Occupational risk of spotted fever: an evaluation of knowledge, attitudes and prevention practices among veterinary medicine students. Rev Patol Trop. 2014;43(4):389−97.

[65] Mallawarachchi C, Gunaratne I, Ekanayaka G, Mallawarachchi S, Chandrasena T, Mendis D, et al. Detection of a case of Brugian Filariasis from Anuradhapura, a non-endemic district of Sri Lanka. Anniversary Academic Sessions; 2018; Colombo, Sri Lanka: Sri Lanka Medical Association; 2018.

[66] Chandrasena NTGA, Premaratna R, Samarasekera D, de Silva N. Surveillance for transmission of lymphatic filariasis in Colombo and Gampaha districts of Sri Lanka following mass drug administration. Trans R Soc Trop. 2016;110(10):620−2. doi: 10.1093/trstmh/trw067 27816936

[67] Weerathunga D, Amarasinghe A, Iddawela D, Wickramasinghe S. Prevalence of canine tick-borne haemoparasites in three divisional secretariat divisions (Rambewa, Tirappane, and Galenbidunuwewa) in the Anuradhapura district, Sri Lanka. Sri Lankan Journal of Infectious Diseases. 2019;9(2).

[68] Sawford K, Vollman AR, Stephen C. A focused ethnographic study of Sri Lankan government field veterinarians’ decision making about diagnostic laboratory submissions and perceptions of surveillance. PLOS One. 2012;7(10):e48035. doi: 10.1371/journal.pone.0048035 23133542 PMC3485039

[69] Desquesnes M, Holzmuller P, Lai DH, Dargantes A, Lun ZR, Jittaplapong S. *Trypanosoma evansi* and surra: a review and perspectives on origin, history, distribution, taxonomy, morphology, hosts, and pathogenic effects. Biomed Res Int. 2013;2013:194176. 24024184 10.1155/2013/194176 PMC3760267

[70] European Scientific Counsel Companion Animal Parasites (ESCCAP). ESCCAP Guideline 05. Malvern Hills Science Park, Geraldine Road, Malvern, Worcestershire, WR14 3SZ, United Kingdom: ESCCAP; 2023.

[71] Checa R, Montoya A, Ortega N, Gonzalez-Fraga JL, Bartolome A, Galvez R, et al. Efficacy, safety and tolerance of imidocarb dipropionate versus atovaquone or buparvaquone plus azithromycin used to treat sick dogs naturally infected with the *Babesia microti*-like piroplasm. Parasit Vectors. 2017;10(1):145. 28292316 10.1186/s13071-017-2049-0 PMC5404670

[72] Han D, Yoon WK, Hyun C. Cerebellar encephalopathy from diminazene aceturate (beneril) toxicity in a dog. Korean Journal of Veterinary Research. 2014;54(3):193–6.

[73] Obeyesekere N. Needs, difficulties, and possible approaches to providing quality clinical veterinary education with the aim of improving standards of companion animal medicine in sri lanka. J Vet Med Educ. 2004;31(1):32−7. doi: 10.3138/jvme.31.1.32 15962247

[74] Rathish D, Rajapakse J, Weerakoon K. Household preferences for pet keeping: Findings from a rural district of Sri Lanka. Plos One. 2022;17(11):e0277108. doi: 10.1371/journal.pone.0277108 36413533 PMC9681089

[75] National rabies control program. Statistical Bulletin. Ministry of Health, Sri Lanka, services Phv; 2020.

[76] Klein GE. Dr. Girl: The feminisation of veterinary medicine [Ph.D.]. United States—Georgia: Georgia State University; 2002.

[77] Irvine L, Vermilya JR. Gender work in a feminised profession: The case of veterinary medicine. Gender & Society. 2010;24(1):56−82.

[78] Ahamed Lebbe S. Gender Imbalances in University Admission in Sri Lanka. Journal of Management. 2011;VII(1):24–35.

[79] Department of Census & Statistics- Sri Lanka. Undergraduate enrolment by academic programme and sex 2021 [Available from: http://www.statistics.gov.lk/GenderStatistics/StaticalInformation/Education.

[80] Colella V, Wongnak P, Tsai Y-L, Nguyen V-L, Tan DY, Tong KBY, et al. Human social conditions predict the risk of exposure to zoonotic parasites in companion animals in East and Southeast Asia. Communications Medicine. 2022;2(1):144. doi: 10.1038/s43856-022-00210-8 36380151 PMC9666534

[81] Lashnits EW, Dawson DE, Breitschwerdt E, Lanzas C. Ecological and socioeconomic factors associated with *Bartonella henselae* exposure in dogs tested for vector-borne diseases in North Carolina. Vector Borne Zoonotic Dis. 2019;19(8):582−95. 31112095 10.1089/vbz.2018.2397 PMC6685192

