# Supplementary material for: Knowledge, attitude, and practices of veterinarians towards canine vector-borne pathogens in Sri Lanka
Source: PLoS Negl Trop Dis. 2024 Jul 29;18(7):e0012365. doi: 10.1371/journal.pntd.0012365 (PMC11309419; doi:10.1371/journal.pntd.0012365)
Supplement: S1 File — (PDF) [file pntd.0012365.s001.pdf]

Questionnaire for veterinarians on canine vector-borne pathogen infections in Sri Lanka

1 Are you a registered to practice lawfully as a veterinarian in Sri Lanka under the Veterinary Surgeons and Practitioners Act, No. 46 of 1956?
☐ Yes
☐ No [End survey]

2 Are you currently residing AND working in Sri Lanka?
☐ Yes
☐ No [End survey]

3 Are you currently working as a veterinarian or in a veterinary or a public health related field?
☐ Yes
☐ No [End survey]

Your background

4 From which veterinary college did you obtain your degree?
☐ University of Peradeniya
☐ Other ………………

5 What is your age group (years)?
☐ 18-24
☐ 25-34
☐ 35- 44
☐ 45-54
☐ ≥ 55

6 What is your gender?
☐ Female
☐ Male
☐ Other
☐ I prefer not to say

Information about your discipline and practice

7 What is the primary area that you are working in?
☐ Academia and research
☐ Aquaculture and fish
☐ Companion/Small animal practice
☐ Farm/Large animal practice
☐ Government
☐ Pharmaceuticals
☐ Poultry
☐ Public health
☐ Wildlife and exotics
☐ Other, please specify …………….

8 What species do you routinely encounter in your practice/work?
☐ Cat
☐ Cattle
☐ Dog
☐ Elephant
☐ Equine
☐ Goat and sheep
☐ Poultry and other avian species
☐ Swine
☐ Wildlife
☐ Other, please specify…………………
☐ None

10 Approximately what proportion of your daily number of consultations are canine consultations?

☐ Less than 25%
☐ Around 25-50%
☐ Around 51-75%
☐ More than 75%

11 How would you rate your overall knowledge of canine vector-borne diseases (e.g., tick fever, filariasis, trypanosomiasis)?

☐ Very poor
☐ Poor
☐ Satisfactory
☐ Good
☐ Excellent

12 How do you keep up to date on diagnosis, treatment, management, and control of canine vector-borne diseases?

☐ Attending face-to-face workshops and seminars
☐ Attending webinars
☐ Scientific publications
☐ Social media
☐ Sources available on the internet
☐ Talking to colleagues
☐ Talking to academics and other teachers
☐ Veterinary and related textbooks
☐ Other, please specify..........................
☐ I do not keep myself updated

13 Canine vector-borne diseases are caused by viruses, bacteria and parasites that are transmitted through arthropod hosts − ticks, fleas, mosquitoes, and flies − referred to as 'vectors'.

☐ True
☐ False
☐ Don't know

14 The definitive diagnosis of most of the canine vector-borne diseases can be ascertained by physical examination of the patient alone.

☐ True
☐ False
☐ Don't know

15 'Tick fever' is a syndrome caused by pathogens such as *Babesia vogeli, Babesia gibsoni, Hepatozoon canis, Ehrlichia canis* and *Anaplasma platys*.

☐ True
☐ False
☐ Don't know

16 Failure to observe a pathogen responsible for tick fever on a blood smear, rules out the disease in that patient.

☐ True
☐ False
☐ Don't know

17 Treatment for vector-borne diseases in dogs always results in complete elimination of the pathogen.

☐ True
☐ False
☐ Don't know

Please select the common vector responsible for transmitting the following pathogens to dogs in Sri Lanka.

18 *Babesia vogeli*
- ☐ Fleas
- ☐ Mosquitoes
- ☐ Ticks
- ☐ Other flies
- ☐ Don't know

19 *Dirofilaria repens*
- ☐ Fleas
- ☐ Mosquitoes
- ☐ Ticks
- ☐ Other flies
- ☐ Don't know

20 *Trypanosoma evansi*
- ☐ Fleas
- ☐ Mosquitoes
- ☐ Ticks
- ☐ Other flies
- ☐ Don't know

Please indicate whether the following vector-borne pathogens in dogs are zoonotic (i.e. transmissible between animals and humans).

21 *Babesia gibsoni*
- ☐ Yes, zoonotic
- ☐ No, not zoonotic
- ☐ Don't know

22 *Dirofilaria repens*
- ☐ Yes, zoonotic
- ☐ No, not zoonotic
- ☐ Don't know

23 *Hepatozoon canis*
- ☐ Yes, zoonotic
- ☐ No, not zoonotic
- ☐ Don't know

24 **Which of the following vector-borne pathogens do you think are reported in Sri Lanka?**
- ☐ *Anaplasma phagocytophilum*
- ☐ *Anaplasma platys*
- ☐ *Babesia canis*
- ☐ *Babesia gibsoni*
- ☐ *Babesia vogeli*
- ☐ *Bartonella henselae*
- ☐ *Brugia* spp.
- ☐ *Dirofilaria immitis*
- ☐ *Dirofilaria repens*
- ☐ *Ehrlichia canis*
- ☐ Haemotrophic mycoplasma spp.
- ☐ *Hepatozoon canis*

☐ *Leishmania* spp.
☐ *Rickettsia conorii*
☐ *Trypanosoma evansi*

Please indicate your level of agreement with the following statements.

25	Sri Lankan veterinarians can manage and treat vector-borne diseases in dogs efficiently and effectively compared to veterinarians in more economically advanced countries like Canada, the United States of America, Australia, or Japan.	☐ Strongly disagree<br>☐ Disagree<br>☐ Neutral<br>☐ Agree<br>☐ Strongly agree
26	It is important for veterinarians to be vigilant about vector-borne diseases that can be transmitted from dogs to humans.	☐ Strongly disagree<br>☐ Disagree<br>☐ Neutral<br>☐ Agree<br>☐ Strongly agree
27	It is important to have good surveillance of vector-borne diseases in dogs in Sri Lanka.	☐ Strongly disagree<br>☐ Disagree<br>☐ Neutral<br>☐ Agree<br>☐ Strongly agree
28	Effective ectoparasite control is the best approach for the prevention of tick fever in dogs.	☐ Strongly disagree<br>☐ Disagree<br>☐ Neutral<br>☐ Agree<br>☐ Strongly agree
29	A definitive diagnosis is necessary for the effective treatment of vector-borne diseases in dogs	☐ Strongly disagree<br>☐ Disagree<br>☐ Neutral<br>☐ Agree<br>☐ Strongly agree

How often do you diagnose diseases caused by the following vector-borne pathogens in dog?

30	Tick fever	☐ Never<br>☐ Yearly<br>☐ Monthly<br>☐ Weekly<br>☐ Daily
31	Filariosis	☐ Never<br>☐ Yearly<br>☐ Monthly<br>☐ Weekly<br>☐ Daily

32	Hepatozoonosis	☐ Never<br>☐ Yearly<br>☐ Monthly<br>☐ Weekly<br>☐ Daily
33	Trypanosomiasis	☐ Never<br>☐ Yearly<br>☐ Monthly<br>☐ Weekly<br>☐ Daily

On average, what is the prognosis of dogs with the following disease in your practice?

34	Tick fever	☐ Grave<br>☐ Poor<br>☐ Fair<br>☐ Good<br>☐ Excellent<br>☐ Don't know
35	Filariosis	☐ Grave<br>☐ Poor<br>☐ Fair<br>☐ Good<br>☐ Excellent<br>☐ Don't know
36	Hepatozoonosis	☐ Grave<br>☐ Poor<br>☐ Fair<br>☐ Good<br>☐ Excellent<br>☐ Don't know
37	Trypanosomiasis	☐ Poor<br>☐ Fair<br>☐ Good<br>☐ Excellent<br>☐ Don't know

Select the most appropriate option based on your practice

38	I base my diagnosis of a canine vector-borne disease on the dog's clinical signs.	☐ Never<br>☐ Rarely<br>☐ Sometimes<br>☐ Very often<br>☐ Always
39	I base my diagnosis of canine vector-borne diseases on clinical signs and other diagnostic aids (e.g., FBC, Blood smear examination, serology).	☐ Never<br>☐ Rarely<br>☐ Sometimes<br>☐ Very often

☐ Always

40 When I diagnose a dog with babesiosis, I also treat for potential tick-borne co-infections (e.g., ehrlichiosis).

☐ Never
☐ Rarely
☐ Sometimes
☐ Very often
☐ Always

41 Monetary constrains hinder proper diagnosis and treatment of canine vector-borne diseases in Sri Lanka.

☐ Never
☐ Rarely
☐ Sometimes
☐ Very often
☐ Always

42 I inform my clients on the importance of tick control for their pet dogs.

☐ Never
☐ Rarely
☐ Sometimes
☐ Very often
☐ Always

Your treatment choices

43 What is your drug/drug-combination of choice to treat infections with *Babesia gibsoni* in dogs?
☐ Diminazene aceturate (Berenil)
☐ Doxycycline
☐ Imidocarb dipropionate
☐ Metronidazole, Clindamycin, and Doxycycline combination
☐ Other, please specify…………………………………
☐ I don't treat *Babesia gibsoni*

44 What is your drug/drug-combination of choice to treat infections with *Babesia vogeli* (syn. *B. canis*) in dogs?
☐ Diminazene aceturate (Berenil)
☐ Doxycycline
☐ Imidocarb dipropionate
☐ Metronidazole, Clindamycin, and Doxycycline combination
☐ Other, please specify……………………….
☐ I don't treat *Babesia vogeli*

45 What is your drug/drug-combination of choice to treat infections with *Ehrlichia canis* in dogs?
☐ Diminazene aceturate (Berenil)
☐ Doxycycline
☐ Imidocarb dipropionate
☐ Metronidazole, Clindamycin, and Doxycycline combination
☐ Other, please specify……………………….
☐ I don't treat *Ehrlichia canis*

46 What is your drug/drug-combination of choice to treat infections with *Dirofilaria repens* in dogs?
☐ Ivermectin or other macrocyclic lactones
☐ Levamisole
☐ Other, please specify………………………………..
☐ I don't treat *Dirofilaria repens*

From the list of ectoparasiticides given below, indicate how likely you are to recommend the following products in your practice to treat and/or control tick and flea infestation in adult dogs.

47 Fipronil, e.g., Frontline spot-on/spray, Tix-free spot-on

☐ Never
☐ Rarely
☐ Sometimes
☐ Very often
☐ Always

48 Propoxur e.g., Bolfo powder

☐ Never
☐ Rarely
☐ Sometimes
☐ Very often
☐ Always

49 Amitraz e.g., Soltic dog collar, Tikamit solution, Taktic solution

☐ Never
☐ Rarely
☐ Sometimes
☐ Very often
☐ Always

50 Isoxazoline compounds e.g., NexGard, Bravecto, Simparica

☐ Never
☐ Rarely
☐ Sometimes
☐ Very often
☐ Always

51 Pyrethrin/ synthetic pyrethroids e.g., Kawu shampoo, Tixfree shampoo, Butox vet, Bayticol, Can Can shampoo

☐ Never
☐ Rarely
☐ Sometimes
☐ Very often
☐ Always

52 Macrocyclic lactones e.g., Ivermectin, Abamectin, Doramectin

☐ Never
☐ Rarely
☐ Sometimes
☐ Very often
☐ Always

53 Natural/ herbal products e.g., herbal pet soaps and shampoos, (Scooby dog, Fur up), Herbal powders (Erina-Himalaya), Herbal oils and extracts (margosa oil, neem oil)

☐ Never
☐ Rarely
☐ Sometimes
☐ Very often
☐ Always

24 Other, please specify………………

☐ Never
☐ Rarely
☐ Sometimes
☐ Very often
☐ Always

[End survey]
