# Supplementary material for: Knowledge, attitude, and practices of veterinarians towards canine vector-borne pathogens in Sri Lanka
Source: PLoS Negl Trop Dis. 2024 Jul 29;18(7):e0012365. doi: 10.1371/journal.pntd.0012365 (PMC11309419; doi:10.1371/journal.pntd.0012365)
Supplement: S1 Table — (PDF) [file pntd.0012365.s003.pdf]

S1 Table. Responses provided by veterinarians in Sri Lanka (n = 170) to knowledge evaluation statements regarding canine vector-borne pathogens. Correct responses are indicated for each statement.

**Statement**	**Correct n (%)**	**Incorrect n (%)**	**Don't know n (%)**
Canine vector-borne diseases are caused by viruses, bacteria and parasites that are transmitted through arthropod hosts − ticks, fleas, mosquitoes, and flies − referred to as 'vectors'.<br>Correct answer: Yes.	152 (89.4)	17 (10)	1 (0.6)
The definitive diagnosis of most of canine vector-borne diseases can be ascertained by physical examination of the patient alone.<br>Correct answer: No.	149 (87.6)	20 (11.8)	1 (0.6)
'Tick fever' is a syndrome caused by pathogens such as *Babesia vogeli, Babesia gibsoni*, *Hepatozoon canis*, *Ehrlichia canis* and *Anaplasma platys.*<br>Correct answer: Yes.	148 (87.1)	21 (12.4)	1 (0.6)
Failure to observe a pathogen responsible for tick fever on a blood smear rules out the disease in that patient.<br>Correct answer: No.	156 (91.8)	11 (6.5)	3 (1.8)
Treatment for vector-borne diseases in dogs always results in the complete elimination of the pathogen.<br>Correct answer: No.	159 (93.5)	9 (5.3)	2 (1.2)
**Knowledge of the usual vector responsible for transmitting the following pathogens to dogs in Sri Lanka:**			
Babesia vogeli - Correct answer: transmitted by ticks	148 (87.1)	6 (3.5)	16 (9.4)
Dirofilaria repens - Correct answer: transmitted by mosquitoes	157 (92.4)	10 (5.9)	3 (1.8)
Trypanosoma evansi - Correct answer: transmitted by other flies	89 (52.4)	65 (38.2)	16 (9.4)
**Knowledge of whether the following canine vector-borne pathogens are zoonotic:**			
Babesia gibsoni - Correct answer: No, not zoonotic	145 (85.3)	18 (10.6)	7 (4.1)
Dirofilaria repens - Correct answer: Yes, zoonotic	98 (57.6)	63 (37.1)	9 (5.3)
Hepatozoon canis - Correct answer: No, not zoonotic	136 (80)	20 (11.8)	14 (8.2)
