# Supplementary material for: Knowledge, attitude, and practices of veterinarians towards canine vector-borne pathogens in Sri Lanka
Source: PLoS Negl Trop Dis. 2024 Jul 29;18(7):e0012365. doi: 10.1371/journal.pntd.0012365 (PMC11309419; doi:10.1371/journal.pntd.0012365)
Supplement: S2 Table — (PDF) [file pntd.0012365.s004.pdf]

S2 Table. Vector-borne pathogens endemic to Sri Lanka according to the scientific literature up until December 2021, and a summary of responses obtained from 170 Sri Lankan veterinarians through a knowledge, attitude, and practices survey around canine vector-borne pathogens.

**Vector-borne pathogen**	**Presence (P) or absence (A)**[†]	**Correct responses n (%)**	**Reference**
Ehrlichia canis	P	165 (97.1)	(1-3)
Babesia gibsoni	P	164 (96.5)	(3-5)
Babesia canis	P	159 (93.5)	(6)
Hepatozoon canis	P	156 (91.8)	(7, 8)
Bartonella henselae	A	150 (88.2)	
Babesia vogeli	A	148 (87.1)	(6)
Anaplasma phagocytophilum	A	146 (85.9)	
Leishmania spp.	P	139 (81.8)	(9, 10)
Dirofilaria repens	P	128 (75.3)	(11)
Trypanosoma evansi	P	121 (71.2)	(12)
Dirofilaria immitis	A	98 (57.6)	
Anaplasma platys	P	54 (31.8)	(13)
Rickettsia conorii	P	31 (18.2)	(14, 15)
Brugia spp.	P	27 (15.9)	(11)
Haemotropic mycoplasmas	P	20 (11.8)	(16)

[†]including published reports up until December 2021

References

1. McGaughey CA, Seneviratna P, Mahalingam S. Rickettsiosis of dogs in Ceylon. Ceylon Veterinary Journal. 1962;10(2-3):82-7 pp.

2. Bennett SR, Quinn RL, Teleford S, Rich S, Kulasekera V, Obeysekera N, et al. The prevalence and significance of canine ehrlichiosis in Colombo, Sri Lanka. Sri Lanka Veterinary Journal. 2005;52:1-8.

3. Weerathunga D, Amarasinghe A, Iddawela D, Wickramasinghe S. Prevalence of canine tick-borne haemoparasites in three divisional secretariat divisions (Rambewa, Tirappane, and Galenbidunuwewa) in the Anuradhapura district, Sri Lanka. Sri Lankan Journal of Infectious Diseases. 2019;9(2).

4. Senevirathne P. The pathology of *Babesia gibsoni* (Patton, 1910) infection in the dog. Ceylon Veterinary Journal. 1965;8(4):107-10.

5. Senevirathne P. Piroplasmosis of dogs in Ceylon. Ceylon Veterinary Journal. 1953;1:95.

6. Birkenheuer AJ, Buch J, Beall MJ, Braff J, Chandrashekar R. Global distribution of canine *Babesia* species identified by a commercial diagnostic laboratory. Veterinary Parasitology: Regional Studies and Reports. 2020;22:100471.

7. Dissanaike A. *Hepatozoon canis* infection in dogs in Ceylon. Ceylon Veterinary Journal. 1961;IX:144-5.

8. Atapattu U, Dissanayake ADR, Silva ID, Bulumulla DGSS, Neelwala NGDAK, Wijekoon T. Acute heaptozoonosis caused by *Hepatozoon canis* in dogs in Sri Lanka. Sri Lanka Veterinary Journal. 2017;64(1A).

9. Karunaweera ND, Ginige S, Senanayake S, Silva H, Manamperi N, Samaranayake N, et al. Spatial Epidemiologic Trends and Hotspots of Leishmaniasis, Sri Lanka, 2001-2018. Emerg Infect Dis. 2020;26(1):1-10.

10. Siriwardana Y, Deepachandi B, Weliange SS, Udagedara C, Wickremarathne C, Warnasuriya W, et al. First evidence for two independent and different leishmaniasis transmission foci in Sri Lanka: Recent Introduction or Long-Term Existence? J Trop Med. 2019;2019:6475939.

11. Mallawarachchi CH, Chandrasena NTGA, Wickramasinghe S, Premaratna R, Gunawardane NYIS, Mallawarachchi NSMSM, et al. A preliminary survey of filarial parasites in dogs and cats in Sri Lanka. PLoS One. 2018;13(11):e0206633.

12. Dangolla A, Wijesundara DLR, Blair D, Fernando DD, Wijesundera KK, Chathuranga WGD, et al. Canine trypanosomosis in Sri Lanka: An emerging problem reported from three distinct geographic locations. Parasitology international. 2020;77:102129.

13. Wijeratne HSU, Chamari GDL, Chathurangi AGM, Perera SVN. Prevalance of *Anaplasma* spp in anemic dogs presented to a veterinary hospital in Kalutara. 72nd Annual Scientific Sessions of the Sri Lanka Veterinary Association; 2020; Faculty of Veterinary Medicine and Animal Sciences, University of Peradeniya.

14. Stokes PH, Walters BJ. Spotted fever rickettsiosis infection in a traveler from Sri Lanka. J Travel Med. 2009;16(6):436-8.

15. Weerakoon K, Kularatne SAM, Rajapakse J, Adikari S, Waduge R. Cutaneous manifestations of spotted fever rickettsial infections in the Central Province of Sri Lanka: A descriptive study. PLoS Neglected Tropical Diseases. 2014;8(9):e3179.

16. Seneviratna P, Weerasinghe, Ariyadasa S. Transmission of *Haemobartonella canis* by the dog tick, *Rhipicephalus sanguineus*. Res Vet Sci. 1973;14(1):112-4.
