# Supplementary material for: Knowledge, attitude, and practices of veterinarians towards canine vector-borne pathogens in Sri Lanka
Source: PLoS Negl Trop Dis. 2024 Jul 29;18(7):e0012365. doi: 10.1371/journal.pntd.0012365 (PMC11309419; doi:10.1371/journal.pntd.0012365)
Supplement: S3 Table — (PDF) [file pntd.0012365.s005.pdf]

S3 Table. Responses of canine practitioners in Sri Lanka (n= 128) on diagnosis, treatment, control, and the prognosis of CVBP infections in dogs.

Variable	Category	n	%
**Frequency of encountering cases**†			
Tick fever (n=126)	Never	0	0
	Yearly	1	0.8
	Monthly	9	7.1
	Weekly	35	27.8
	Daily	81	64.3
Filariasis (n=126)	Never	2	1.6
	Yearly	22	17.5
	Monthly	31	24.6
	Weekly	30	23.8
	Daily	41	32.5
Hepatozoonosis (n=127)	Never	25	19.8
	Yearly	36	28.6
	Monthly	47	37.3
	Weekly	16	12.7
	Daily	2	1.6
Trypanosomiasis (n=127)	Never	65	51.6
	Yearly	49	38.9
	Monthly	11	8.7
	Weekly	1	0.8
	Daily	0	0
**Perceived prognosis**†			
Tick fever (n=123)	Grave	1	0.8
	Poor	0	0
	Fair	5	4.1
	Good	76	61.8
	Excellent	41	33.3
	Don't know	0	0
Filariasis (n=122)	Grave	1	0.8
	Poor	1	0.8
	Fair	19	15.6
	Good	58	47.5
	Excellent	41	33.6
	Don't know	2	1.7
Hepatozoonosis (n=123)	Grave	1	0.8
	Poor	9	7.3
	Fair	27	22

	Good	49	39.8
	Excellent	16	13
	Don't know	21	17.1
Trypanosomiasis (n=123)	Grave	2	1.6
	Poor	13	10.6
	Fair	22	17.9
	Good	24	19.5
	Excellent	2	1.6
	Don't know	60	48.8
**Diagnosis**			
Based on clinical signs (n=118)	Never or rarely	17	14.4
	Sometimes	50	42.4
	Very often or always	51	43.2
Based on clinical signs and other diagnostic aids (n=118)	Never or rarely	8	6.8
	Sometimes	32	27.1
	Very often or always	78	66.1
Monetary constraints (n=118)	Never or rarely	12	10.2
	Sometimes	68	57.6
	Very often or always	38	32.2
**Treatment**‡			
Babesia gibsoni (n=118)	Imidocarb dipropionate	50	42.4
	Diminazene aceturate	42	35.6
	Metronidazole, Clindamycin & Doxycycline	12	10.2
	Doxycycline	7	5.9
	Atovaquone & Azithromycin	4	3.4
	Other§	3	2.5
Babesia vogeli (n=118)	Imidocarb dipropionate	56	47.5
	Diminazene aceturate	28	23.7
	Doxycycline	9	7.6
	Metronidazole, Clindamycin & Doxycycline	9	7.6
	Other¶	2	1.7
	Not treating	14	11.9
Ehrlichia canis (n =118)	Doxycycline	78	66.1
	Imidocarb dipropionate	34	28.8
	Metronidazole, Clindamycin & Doxycycline	3	2.5
	Diminazene aceturate	2	1.7
	Other#	1	0.9
Dirofilaria repens (n = 118)	Macrocyclic lactones	59	50

	Levamisole	49	41.5
	Diethylcarbamazine	2	1.7
	Other♠	3	2.5
	Not treating	5	4.3
Co-infections (n = 118)	Never	0	0
	Rarely	6	5.1
	Sometimes	35	29.7
	Very often	55	46.6
	Always	22	18.6
**Prevention and control**‡			
Informing dog owners on ectoparasite control (n=118)	Never	0	0
	Rarely	0	0
	Sometimes	1	0.8
	Very often	17	14.4
	Always	100	84.8
Recommendation of the following products to treat and/or control tick and flea infestation in adult dogs:			
Fipronil (n=116)	Never	0	0
	Rarely	6	5.2
	Sometimes	22	19
	Very often	75	64.6
	Always	13	11.2
Propoxur (n=116)	Never	4	3.4
	Rarely	19	16.4
	Sometimes	34	29.3
	Very often	53	45.7
	Always	6	5.2
Amitraz (n=116)	Never	8	6.9
	Rarely	26	22.4
	Sometimes	53	45.7
	Very often	23	19.8
	Always	6	5.2
Isoxazoline (n=116)	Never	6	5.2
	Rarely	12	10.3
	Sometimes	25	21.6
	Very often	49	42.2
	Always	24	20.7
Pyrethrin (n=116)	Never	7	6
	Rarely	32	27.6
	Sometimes	54	46.6
	Very often	19	16.4

	Always	4	3.4
Macrocyclic lactones (n=116)	Never	20	17.1
	Rarely	44	37.6
	Sometimes	34	29.1
	Very often	15	12.8
	Always	4	3.4
Herbal (n=116)	Never	18	15.5
	Rarely	32	27.6
	Sometimes	50	43.1
	Very often	15	12.9
	Always	1	0.9
Other[♥] (n=114)	Never	92	80.7
	Rarely	10	8.7
	Sometimes	10	8.7
	Very often	1	0.9
	Always	1	0.9
**CVBD practice score (n =118)**	Low - moderate	64	54.2
	High	54	45.8

[†]Reported based on veterinarians' responses to the survey, and might not reflect the actual scenario
[‡]The treatment/preventive options listed herein are a summary of responses to the survey, which may include recommended as well as non-recommended treatment/preventive options for respective pathogens.
[§]Atovaquone/proguanil; atovaquone only; imidocarb dipropionate with metronidazole, doxycycline, and clindamycin combination.
[¶]Atovaquone; imidocarb dipropionate with metronidazole, doxycycline, and clindamycin combination.
[#]Imidocarb dipropionate with metronidazole, doxycycline, and clindamycin combination.
[♠]Doxycycline; macrocyclic lactones and levamisole
[♥]Electric flea comb, manual removal, cleaning the environment.
