# Supplementary material for: Knowledge, attitude, and practices of veterinarians towards canine vector-borne pathogens in Sri Lanka
Source: PLoS Negl Trop Dis. 2024 Jul 29;18(7):e0012365. doi: 10.1371/journal.pntd.0012365 (PMC11309419; doi:10.1371/journal.pntd.0012365)
Supplement: S4 Table — (PDF) [file pntd.0012365.s006.pdf]

S4 Table. Demographic factors, confidence in profession and self-rated knowledge associated with knowledge around canine vector-borne pathogen (CVBP) infections determined through univariable ordinal regression based on responses of veterinarians in Sri Lanka for a knowledge, attitude, and practices survey.

**Variable**<br>**Categories**	**Estimate**	**SE**	**Odds ratio (95% CI)**	**P-value**
**Gender (n = 166)**				<0.001
Female	1.08	0.3	3 (1.6 – 5.4)	
Male			Reference	
**Age group in years (n=170)**				0.03
25 - 34	1.21	0.47	3.4 (1.4 – 8.6)	
35 - 44	0.72	0.57	2 (0.7 – 6.4)	
≥ 45			Reference	
**Experience (n= 170)**				0.021
New graduates	1.62	0.59	5.1 (1.7 – 17.4)	
Moderately experienced	0.36	0.31	1.4 (0.8 – 2.7)	
Experienced			Reference	
**Primary discipline of practice (n=170)**				0.03
Academia	0.74	0.42	2.1 (0.9 – 4.9)	
Companion animal practice	0.31	0.33	1.4 (0.7 – 2.6)	
Government			Reference	
Other	-1.07	0.58	0.3 (0.1 – 1.1)	
**Canine caseload (n = 170)**				0.309
No - moderate			Reference	
High	0.53	0.35	1.7 (0.87 – 3.36)	
Very high	0.16	0.35	1.2 (0.59 – 2.36)	
**No. of information sources (n = 170)**				0.092
≤ 3			Reference	
4 - 5	0.21	0.31	1.2 (0.7 – 2.3)	
≥ 6	0.92	0.42	2.5 (1.1 – 5.8)	
**Confidence in profession (n = 167)**				0.761
Low	0.33	0.46	1.4 (0.6 – 3.5)	
Moderate	0.05	0.31	1 (0.6–1.9)	
High			Reference	
**Self-rated knowledge (n = 170)**				0.178
Low-moderate			Reference	
High	0.39	0.29	1.5 (0.8 – 2.6)	

CI = confidence interval, SE = standard error
