# Supplementary material for: Knowledge, attitude, and practices of veterinarians towards canine vector-borne pathogens in Sri Lanka
Source: PLoS Negl Trop Dis. 2024 Jul 29;18(7):e0012365. doi: 10.1371/journal.pntd.0012365 (PMC11309419; doi:10.1371/journal.pntd.0012365)
Supplement: S5 Table — (PDF) [file pntd.0012365.s007.pdf]

S5 Table. Associations of knowledge, practice type, confidence in profession, experience, canine caseload, and monetary constraints with practices on diagnosis, treatment, and control of canine vector-borne pathogen infections determined through univariable logistic regression based on responses of canine practitioners in Sri Lanka for a knowledge, attitude, and practices survey.

Variable<br>Categories	n	Estimate	SE	Odds ratio (95% CI)	P-value
**Gender (n = 115)**					**0.923**
Female	75	0.04	0.38	1 (0.5 - 2.2)	
Male	50			Reference	
**Age group in years (n=118)**					**0.938**
25 - <35	97			Reference	
≥35	31	-0.03	0.42	1 (0.4 - 2.2)	
**Experience (n = 118)**					**0.651**
New graduates or moderately experienced	117			Reference	
Experienced	53	0.18	0.39	1.2 (0.6 - 2.6)	
**Primary discipline of practice (n=118)**					**0.141**
Companion animal practice	65	0.55	0.37	1.7 (0.8 - 3.6)	
Other[†]	63			Reference	
**Canine caseload (n=118)**					**0.122**
Low - moderate	38	0.94	0.48	2.6 (1 - 6.8)	
High	49	0.23	0.45	1.3 (0.5 - 3.1)	
Very high	41			Reference	
**No. of information sources (n = 118)**					**0.010**
≤ 3	50			Reference	
4	31	0.74	0.5	2.1 (0.8- 5.8)	
≥ 5	47	-0.83	0.44	0.4 (0.2 - 1)	
**Confidence in profession (n = 118)**					**0.965**
Low - moderate	76			Reference	
High	51	0.02	0.37	1 (0.5 - 2.1)	
**Actual knowledge (n =118*)***					**0.805**
Low - moderate	72			Reference	
High	56	0.09	0.37	1.1 (0.5 - 2.3)	
**Self-rated knowledge (n = 118)**					**0.697**
Low-moderate	54	0.15	0.38	1.2 (0.6 - 2.4)	
High	74			Reference	
**Agreement between self-rated vs actual knowledge (n =118)**					**0.688**
Low - moderate	62			Reference	
High	66	0.15	0.37	1.2 (0.6 - 2.4)	
**Monetary constraints (n =118)**					**0.525**
Never - sometimes	80			Reference	
Very often or always	38	0.25	0.4	1.3 (0.6 - 2.8)	

[†]Academia, farm animals, government, poultry, wildlife, and exotics
CI = confidence interval; SE = standard error
