# Supplementary material for: Knowledge, attitude, and practices of veterinarians towards canine vector-borne pathogens in Sri Lanka
Source: PLoS Negl Trop Dis. 2024 Jul 29;18(7):e0012365. doi: 10.1371/journal.pntd.0012365 (PMC11309419; doi:10.1371/journal.pntd.0012365)
Supplement: Attachment — Submitted filename: Responses_to_reviewers.pdf [file pntd.0012365.s008.pdf]

RE: Editorial and reviewer comments for "Knowledge, attitude and practices of veterinarians towards canine vector-borne pathogens in Sri Lanka" [PNTD-D-24-00557]

Editorial comments

Editor: The authors have clearly presented the study objectives, methods and process in a way that could be reproduced, which may be helpful in translating this work to other regions impacted by transmission of canine vector-borne pathogens.

Authors: Thank you very much for the positive feedback on our work.

Editor: Please address the reviewer's feedback and provide additional quantitative results supporting the statement (L254-256) around the process of multivariable model selection, specifically identifying the best multivariable model to explain the data, such as how many terms were in the multivariable model(s) prior to backwards-stepwise elimination of variables.

Authors: We added the following to the Results section, to define the terms included in the initial multivariable model:

Lines 251-252 "These variables were included in the initial multivariable ordinal regression model to determine factors associated with CVBP knowledge (**S4 Table**)".

Lines 269-270 "… and were included in the initial multivariable logistic regression model."

The p-values, which indicate the failure to reject the null hypothesis in testing for the proportional odds assumption and the Lipsitz goodness-of-fit test for the model fit, are added to the Results section

Lines 259-L262 "….as the coefficients of the final model from backward step-wise elimination did not change by ≤ 20% when variables were reintroduced. The assumption of proportional odds was fulfilled ($p > 0.05$), and the Lipsitz goodness of fit test indicated a sufficient model fit ($p = 0.79$)."

Lines 276-278 "… as the coefficients of the final model from backward stepwise elimination did not change by ≤ 20% when variables were reintroduced. In addition, diagnostic plots indicated a good model fit."

Please also clarify the description of potential confounding variables and lightly describe why a mixed model regression (e.g. model includes random effects as well as fixed effects) was not used to address anticipated covariance patterns in the data beyond the central variables of interest.

Authors: We modified the following sentence in the Methods section to clarify the description of potential confounding variables:

Lines 154-155 "This would suggest that the variable added substantially influences the relationship between the independent and response variables and thus acts as a confounder."

A mixed model regression including fixed as well as random effects, was not used considering that mix models are more complex and this complexity can make model specification and interpretation more challenging, in particular with lower number of observations as encountered in this study (n = 170). Additionally, the primary focus of this analysis was on modifiable factors while keeping the model accessible and interpretable. By focusing solely on fixed effects, the analysis remains simpler and more straightforward, ensuring clarity in the results and interpretations.

Reviewer comments

2.1 Methods

Reviewer #1: Yes for all the questions

Authors: Thank you

Reviewer #3: Objectives, study design, and population are clearly described and appropriate. The sample size and statistical analysis are adequate to support the hypothesis being tested and the conclusions.

No ethical or regulatory concerns are present.

Authors: Thank you

Just a comment on the period of collection of survey responses (from April to November 2021): overall, 170 completed surveys were collected and included for analysis (8.3% of veterinarians

registered to practice in Sri Lanka). Could this data have been influenced by the pandemic conditions related to SARS-CoV-2 in Sri Lanka in 2021? I recommend discussing in the paper the potential reasons for the moderate adherence of veterinarians to the questionnaire.

Authors: As there is no data available on the current number of practicing veterinarians in Sri Lanka, we calculated the minimum possible proportion responded by considering all veterinarians (n=2041) registered since the 1950s since the establishment of the Veterinary Council of Sri Lanka. This number includes veterinarians who are deceased, no longer practicing, retired, or overseas, and therefore not part of our study population. Therefore, the actual response rate is likely to be much higher, though we are unable to estimate the exact value.

In addition, as mentioned by reviewer#3, COVID-19 potentially influenced the number of veterinarians responded to the questionnaire, and this has now been included in the Discussion section.

Lines 373-374 "…proportion responded. In addition, COVID-19 pandemic related events that took place during the surveying period potentially influenced the number of responses received."

Reviewer #3: However, the study represents an excellent framework of the knowledge on CVBPs by veterinary practitioners in Sri Lanka.

Authors: Thank you for your positive feedback.

2.2 Results

Reviewer #1: Yes for all the questions

Authors: Thank you.

Reviewer #3: Results are clearly and adequately presented; tables and images are useful

Authors: Thank you.

2.3 Conclusions

Reviewer #1: Yes for all the questions

Authors: Thank you.

Reviewer #3: Conclusions are supported by results and Authors discussed adequately their results: this will be useful for implemented continuing veterinary education, considering the public health relevance of CVBP.

Authors: Thank you.

2.4 Editorial and Data Presentation Modifications

Reviewer #1: Abstract

Please change "Canine vector-borne pathogens (CVBP) are highly prevalent " in (CVBP) have a worldwide distribution with higher prevalence ..

Line 18 Please change as above

Authors: Addressed in-text

Line 18 "…have a worldwide distribution and show a high prevalence in tropical countries such as Sri Lanka."

Reviewer #1: Line 59 The role of the dog as a resrvoir for R.conorii is still controversial. *Rhipicephalus sanguineous* is probably the real reservoir, and the dog probably has only a marginal role. Please rephrase this as "Dogs have or may have a role as a reservoir".

Authors: We agree and changed as suggested.

Lines 60-61 "Dogs may have a role as a reservoir for rickettsioses caused by several species of the genus *Rickettsia*…"

Reviewer #3: Line 64: *Babesia vogeli*

Authors: Addressed in-text (**Line 65**).

Reviewer #3: Line 180: *Trypanosoma evansi*

Authors: Addressed in-text (**Line 184**).

Reviewer #3: The acronym KAP is not use at line 281

Authors: Used the acronym in-text (**Line 284**).

2.5 Summary and general comments

Reviewer #1: Very interesting paper that explores the role of the veterinarians and pet care in the control and prevention of vector-borne zoonotic diseases from a "One Health" perspective and how the level of knowledge can depend on various factors.

It may be a starting point for future investigations that compare the levels of knowledge on the same topics by Physicians.

Authors: Thank you for your positive feedback. We also appreciate your interest in our work and your suggestions for future research.

Reviewer #2: Title – replace practice with practices – change accordingly throughout the manuscript

Authors: The title and all relevant sections throughout manuscript were checked, and all relevant places contained practices instead of practice.

Reviewer #2: Keywords – display alphabetically

Authors: Arranged alphabetically in-text

Reviewer #2: Line 21 – adapt to read as: making these pathogens emerging public health ISSUES

Authors: Modified as suggested in-text (**Line 21**).

Reviewer #2: Line 28 – which species of *Brugia*

Authors: As the species of *Brugia* in dogs in Sri Lanka are not adequately characterised, we asked veterinarians in the questionnaire to indicate their awareness of *Brugia* spp. in general to avoid any confusion. Hence, now we changed *Brugia* into *Brugia* spp. (**Line 28**).

Reviewer #2: Line 44 – delete: “by bugs like”

Authors: Text modified as suggested (**Line 45**).

Reviewer #2: Line 48 – replace controlling with preventing

Authors: Replaced with “preventing” as suggested (**Line 47**).

Reviewer #2: Line 52 – adapt: diagnosis, treatment, and prevention of

Authors: Text modified as suggested (**Line 53**).

Reviewer #2: Line 58 – Babesia spp. (instead of Babesia species)

Authors: Text modified as suggested (**Line 59**).

Reviewer #2: Line 62 – adapt: Dirofilaria, WHICH infect

Authors: Text modified as suggested (**Line 63**).

Reviewer #2: Line 64 – write out in full every species name at its first use, e.g. Babesia vogeli

Authors: Text modified as suggested (**Line 65**).

Reviewer #2: Line 72 – delete control or delete treatment

Authors: Text modified as suggested (**Line 73**).

Reviewer #2: Line 103 – these seven volunteers were not included in the final results, right?

Authors: Correct.

Reviewer #2: Line 119 – 11 knowledge questions

Authors: Text modified as suggested **(Line 120**).

Reviewer #2: Line 162 – replace rate with proportion

Authors: Text modified as suggested (**Line 165**).

Reviewer #2: Line 162 – 8.3% is a rather low proportion – please address this issue

Authors: As mentioned in the discussion (**Lines 371-373**), the 8.3% represents the minimum possible proportion of veterinarians in Sri Lanka that could have responded to the survey. This is because the total of 2,041 veterinarians registered with the Veterinary Council of Sri Lanka includes those who are deceased, ill, retired, out of practice, or overseas (**Lines 88-90**). These groups are not relevant to our study population. Therefore, the actual proportion of active veterinarians who responded is likely much higher. However, since we lack the data to determine the exact number of currently practicing veterinarians, we use this value as a rough estimation to depict the representation of the population from the sample obtained, considering it as the minimum possible response rate rather than the actual one.

Reviewer #2: Line 183 – replace However with Nevertheless

Authors: Text modified as suggested (**Line 183**).

Reviewer #2: Line 194 – WERE important.

Authors: Text modified as suggested **(Line 197**).

Reviewer #2: Line 288 – define Illinois and Ohio as USA states

Authors: Text modified as suggested (**Line 291**).

Reviewer #2: Liner 384 – Conclusions

Authors: Text modified as suggested (**Line 388**).

Reviewer #2: Lines 604 and 606 – is there any difference between S1 File and S1 Table?

Authors: Yes, the two files are different. The **S1 File** includes a copy of the questionnaire, while **S1 Table** contains a summary of the responses given to the knowledge questions in the survey.

Reviewer #3: The paper "Knowledge, attitude and practices of veterinarians towards canine vector-borne pathogens in Sri Lanka" describes how veterinarians perceive and address CVBPs in clinical practice. Thought a questionnaire survey, the Authors assessed the KAP of veterinary practitioners and promoted a continuing veterinary education. The scientific soundness is good. The limitations of the study, well described in the text, are correlated to the period of data collection and the unpredictable response rate to the questionnaire.

Authors: Thank you very much for the positive feedback.
